# Genetic Polymorphisms in CD35 Gene Contribute to the Susceptibility and Prognosis of Hepatocellular Carcinoma

**DOI:** 10.3389/fonc.2021.700711

**Published:** 2021-08-05

**Authors:** Limei Luo, Qin Li, Zhenzhen Su, Lixin Li, Bei Cai, Yufu Peng, Yangjuan Bai, Fei Liu

**Affiliations:** ^1^Department of Laboratory Medicine, West China Hospital of Sichuan University, Chengdu, China; ^2^Department of Liver Surgery, Liver Transplantation Center, West China Hospital of Sichuan University, Chengdu, China

**Keywords:** CD35, genetic variation, hepatocellular carcinoma, prognosis, susceptibility

## Abstract

CD35, an important molecule implicated in inflammation and immunity, is reportedly associated with several cancers. However, very few studies have investigated the relationship between *CD35* polymorphisms and hepatocellular carcinoma (HCC). The current study was conducted to investigate the association between tag SNPs in *CD35* and HCC susceptibility and postoperative recurrence, in an attempt to elucidate the gene-environment interactions in HCC. A total of 1233 Chinese Han people, including 647 healthy controls and 586 HCC cases, were sampled in this study. Six Tag SNPs (rs10494885, rs2296160, rs3737002, rs3849266, rs669117, and rs7525160) of *CD35* were selected using the HaploView 4.2 program and genotyped by matrix-assisted laser desorption ionization time-of-flight mass spectrometry (MALDI-TOF-MS). Overall, the mutation genotypes CC/CG of *CD35* rs7525160 significantly increased the risk of HCC. Stratification analysis indicated that *CD35* rs7525160 CC/CG genotypes increased HCC risk in patients younger than 65 years and were closely related to the pathological type of poor prognosis of HCC. Cox proportional hazard ratio model analysis revealed that the rs7525160 CC/CG genotype remains a significant independent risk factor for postoperative recurrence of HCC. In conclusion, *CD35* rs7525160 polymorphism may contribute to the susceptibility and prognosis of HCC in the Chinese Han population.

## Introduction

Hepatocellular carcinoma (HCC), which accounts for more than 80% of primary liver cancers worldwide, is estimated to be the fourth most common cause of cancer-related deaths, and poses a severe disease burden (1, 2). Liver cancer has long dominated all malignancies in China (3), and the cases from China constituted over half of the liver cancer cases (446,100 cases) and deaths (422,100 cases) worldwide in 2015 (4). A multitude of risk factors, including viral factors (HBV and HCV) and non-viral factors, such as alcohol, smoking, aflatoxin, obesity, diabetes, and non-alcoholic fatty liver disease, have been found to be associated with liver cancer (5–9). However, these known risk factors do not fully explain the overall incidence of HCC. Numerous studies have identified many genetic factors that are correlated with HCC susceptibility (10–13). This evidence indicates the consequential role played by genetic backgrounds in the etiology of HCC. Therefore, a better understanding of the molecular mechanisms underlying the initiation and progression of HCC may lead to earlier diagnoses and efficacious therapeutic strategies (14).

The complement system, which has been primarily viewed as the “first line of defense” against microbial intruders, participates in diverse processes, such as clearance of immune complexes, tissue regeneration, and lipid metabolism (15). However, several studies have indicated that the complement system may also promote tumor development in a diverse manner, including sustained cellular proliferation, decreased apoptosis, enhanced invasion, and metastasis (16–18). The liver is the organ, which plays a predominant role in the functioning of the complement system (19). This, in turn, leads to a variety of liver associated effects, such as liver damage, regeneration, and liver transplantation (20).

Complement regulatory proteins comprise a large family that is essential for activation of the complement system (21). One of its members, termed complement receptor 1 (CR1/CD35; hereafter named CD35), is strikingly significant because it interacts not only with C3b and C4b to promote neutrophil-mediated phagocytosis but also plays a role in T-cell and B-cell mediated immune regulation (22, 23). Due to its role in complement activation, innate immunity, and chronic inflammation, CD35 has become a trending molecule in cancer predisposition studies, as attested to by studies that have been conducted on gallbladder cancer, nasopharyngeal carcinoma, non-small cell lung cancer, and gastric cancer (24–27).

However, very few studies have investigated the relationship between *CD35* polymorphisms and HCC predisposition. Therefore, the current case-control study was conducted to investigate the association between tag SNPs in *CD35* and susceptibility to and recurrence of HCC, in an attempt to explore the effect of gene-environment interaction on the risk for HCC.

## Materials and Methods

This study strictly followed all relevant Chinese laws, regulations and rules, as well as the stipulations of the Declaration of Helsinki of the World Medical Association, and was approved by the Ethics Committee of Sichuan University (Ethics No. 2017-264).

### Study Population

Newly diagnosed, untreated HCC patients admitted to the Department of Liver Surgery of the West China Hospital in Sichuan University (Chengdu, China), between May 2017 and March 2019, were enrolled in this study. Diagnosis of HCC was based on histological findings or a combination of elevated serum α-fetoprotein (AFP) and coincident imaging manifestations from two different imaging based diagnostic methods (ultrasonography, computed tomography, or magnetic resonance imaging). Staging criteria for liver cancer were based on Barcelona-Clinic-Liver-Cancer (BCLC) stages (28) and the tumor–node–metastasis (TNM) classification system (29). None of the patients had received radiotherapy or anticancer cytotoxic drug chemotherapy one month prior to blood collection. Patients with previous malignancies or metastasized cancers in other organs, autoimmune diseases, Alzheimer’s disease (AD), and HCC combined pregnancy were excluded. Those who had undergone laparoscopic cholecystectomy for gallstones in the West China Hospital during the same period and had no history of other diseases, tumors, autoimmune diseases or Alzheimer’s disease, were included as healthy controls. All subjects were genetically unrelated Han Chinese, and there were no age or gender restrictions.

Written informed consent was obtained from each participant during recruitment. Baseline characteristics of each subject, such as age, sex, smoking history, drinking history, and family history of HCC, were obtained from the subjects, or their family members, and relevant information on HCC patients, such as tumor size, number of tumors, metastasis, Child–Pugh classification, and detection of microvascular invasion, was further improved by referring to medical records, laboratory tests, and imaging studies.

### Selection of Tag SNPs and Genotype Analysis

SNP genotyping of *CD35* was downloaded from the Human Genome Project and the International HapMap Project. HaploView 4.2 was used to select candidate tag SNPs of *CD35* with an r^2^ threshold of 0.8, and minor allele frequency (MAF) greater than or equal to 0.05, based on Chinese population data from the HapMap database. Finally, six SNPs (rs10494885, rs2296160, rs3737002, rs3849266, rs669117, and rs7525160) were included in this study (**Supplementary Table S1**).

Genomic DNA was extracted from whole blood samples using EDTA anticoagulant *via* a TIANamp Genomic DNA Kit (Tiangen Biotech Co. Ltd., Beijing, China) strictly according to the manufacturer’s instructions. All extracted DNA specimens were diluted to 20 ng/μL in order to obtain a working concentration and stored at -20°C.

Genotyping was carried out using the MassARRAY system (Sequenom, San Diego, CA, USA) *via* the matrix-assisted laser desorption ionization-time of flight mass spectrometry method (MALDI-TOF), according to the manufacturer’s instructions. The detailed genotyping process is described in another study of ours (12). Primer information for selected tag SNPs is shown (**Supplementary Table S2**). Genotyping quality control was performed by verifying genotypic consistency in 5% of samples which were randomly selected and duplicated, while approximately another 5% of the samples were further confirmed *via* direct sequencing.

### HCC Surveillance and Follow-Up

All HCC patients who underwent hepatectomy were regularly followed up in the Outpatient Department of Liver Surgery of the West China Hospital in Sichuan University. During the first year after surgery, the patients were followed up every three months, and the patients with no recurrence for at least 2 years following surgery were followed up every six months instead. Abdominal ultrasonography and blood tests, including liver function tests and AFP levels, were performed at each follow-up. Any subject with a positive finding of AFP or abnormal ultrasonography was subjected to enhanced computed tomography (CT) or magnetic resonance imaging (MRI) of the upper abdomen immediately. Recurrence-free survival (RFS) was defined as the time interval from the date of hepatectomy to the date of recurrence or the deadline for the last follow-up. Overall survival (OS) was defined as the time interval from the date of hepatectomy to the date of death or the last follow-up.

### Statistical Analysis

Statistical analysis was performed using SPSS (version 24.0; IBM Corp., Armonk, NY, USA). All tests were two-tailed, and statistical significance was set at *P*<0.05. Each SNP frequency in controls was analyzed for deviation from the Hardy-Weinberg equilibrium (HWE) using the chi-squared goodness-of-fit test. Mann-Whitney U test (for continuous variables) and chi-square tests with Fisher’s exact test (for categorical variables) were used to compare differences in the distributions of demographic characteristics between the HCC and control groups. The association between HCC risk and *CD35* tag SNPs was estimated as odds ratios (OR) and 95% confidence intervals (CI) using a logistic regression model, adjusted by sex, HBV status, smoking, and drinking status where it was appropriate. The relationship between RFS/OS and *CD35* tag SNPs was analyzed using univariate survival analysis and the Cox proportional hazard ratio model. Family history of liver cancer is defined as family members within three generations of patients with liver cancer. Ever/current smoking is defined as continuous/cumulative smoking for 6 months or more in a lifetime, and consumption of at least 100 cigarettes. Those who had consumed 12 standard drinking amounts (1 standard drinking amount is equal to 10 g of alcohol, approximately 350 ml of regular beer, or about 148 ml of wine, or about 45 ml of 40% white wine) in the past 12 months were defined as ever/current drinkers (30).

## Results

### Patient Characteristics

Initially, 600 HCC cases and 650 cancer-free controls were included in this study. Due to unsuccessful genotyping of 14 HCC cases and 3 controls, a total of 1233 participants consisting of 586 HCC patients (491 men and 95 women) and 647 controls (297 men and 350 women) remained. Baseline characteristics of the HCC cases and controls are summarized (**Table 1**). While there were no significant differences in age (stratified by 65 years) and family history of liver cancer, there were significant differences between gender, hepatitis B, smoking, and drinking status of the two groups (*P*<0.05). A total of 299 HCC patients underwent hepatectomy treatment and were followed up to December 2019, the maximum follow-up time being 30.7 months and a medium follow-up time of 18.0 months. The characteristics and clinical features of HCC patients treated with hepatectomy are summarized (**Supplementary Table S3**).

**Table 1:** Baseline characteristics of HCC patients and controls.

Variable	HCC (n = 586)	Controls (n = 647)	*P*-value
Age [n (%)]			0.092
< 65 years	444 (75.80)	516 (79.80)	
≥ 65 years	142 (24.20)	131 (20.20)	
Gender [n (%)]			< 0.001*
Male	491 (83.80)	297 (45.90)	
Female	95 (16.20)	350 (54.10)	
Smoking status [n (%)]			< 0.001*
Never	275 (46.90)	506 (78.20)	
Ever/current	311 (53.10)	141 (21.80)	
Drinking status [n (%)]			< 0.001*
Never	326 (55.60)	491 (75.90)	
Ever/current	260 (44.40)	156 (24.10)	
Hepatitis B [n (%)]			< 0.001*
With	442 (75.40)	143 (22.10)	
Without	144 (24.60)	504 (77.90)	
Family history of liver cancer [n (%)]			0.347
Without	560 (95.60)	625 (96.60)	
With	26 (4.40)	22 (3.40)	
Child-Pugh Class		NA	
A	500 (93.98)		
B	28 (5.26)		
C	4 (0.76)		
Tumor stage (BCLC)		NA	
0/A	339 (63.84)		
B/C	192 (36.16)		
α-fetoprotein level (ng/mL)		NA	
< 400	418 (71.33)		
≥ 400	168 (28.67)		
Tumor size (cm)		NA	
≤ 5cm	335 (57.17)		
>5cm	251 (42.83)		
Tumor number		NA	
Single	390 (66.55)		
Multiple	196 (33.45)		
Tumor stage (TNM)		NA	
I/II	416 (70.99)		
III/IV	170 (29.01)		
Background cirrhosis		NA	
Present	348 (59.39)		
Absent	238 (40.61)		
Portal vein tumor thrombosis		NA	
No	506 (86.35)		
Yes	80 (13.65)		
Distant metastasis		NA	
No	568 (96.93)		
Yes	18 (3.07)		

*P < 0.05, statically significant. NA, not available.

### Genotype Frequency and Effects of CD35 on HCC Risk

The genotype distribution of *CD35* in controls followed Hardy-Weinberg equilibrium predictions (**Supplementary Table S1**; *P*>0.05). Among the six SNPs of *CD35*, only the frequencies of GG, CG, and CC genotypes of rs7525160 in HCC and control groups were statistically significant (*P*<0.05; **Table 2**). After adjusting for sex, smoking, hepatitis B, and drinking status, the risk of HCC was 1.46-times higher in individuals with at least one mutant allele C (CG+CC) than in individuals with the GG genotype (adjusted OR=1.46; 95% *CI* [1.09-1.95]; *P*=0.012); (**Table 2**). No significant differences were observed between the distribution of rs10494885, rs2296160, rs3737002, rs3849266, and rs6691117 genotypes of the HCC and control groups (*P*>0.05; **Table 2**). The three genotypes of rs7525160 polymorphisms detected by MALDI-TOF and the sequencing map for *CD35* rs7525160 GG and CG genotypes are summarized (**Supplementary Figure S1**).

**Table 2:** SNPs of CD35 gene in patients with HCC and controls.

SNP ID	Genotype	HCC n (%)	Control n (%)	Adjusted OR (95% *CI*)^a^	*P*-value
rs10494885	AA	74 (12.60)	90 (13.90)	1.00 (Reference)	
	AG	251 (42.80)	275 (42.50)	1.159 (0.76, 1.77)	0.493
	GG	261 (44.50)	282 (43.60)	1.131 (0.74, 1.74)	0.575
	GG/AG	512 (87.40)	557 (86.10)	1.156 (0.77, 1.73)	0.479
rs2296160	AA	77 (13.10)	79 (12.20)	1.00 (Reference)	
	AG	240 (41.00)	302 (46.70)	0.84 (0.54, 1.31)	0.443
	GG	269 (50.40)	265 (41.00)	0.98 (0.63, 1.52)	0.922
	GG/AG	509 (86.90)	567 (87.80)	0.91 (0.60, 1.38)	0.652
rs3737002	CC	261 (44.50)	279 (43.10)	1.00 (Reference)	
	CT	245 (41.80)	281 (43.40)	1.09 (0.81, 1.47)	0.571
	TT	80 (13.70)	87 (13.40)	1.02 (0.66, 1.58)	0.917
	TT/CT	325 (55.50)	368 (56.90)	1.07 (0.81, 1.41)	0.639
rs3849266	CC	262 (44.70)	283 (43.90)	1.00 (Reference)	
	CT	244 (41.60)	275 (42.60)	1.11 (0.82, 1.49)	0.506
	TT	80 (13.70)	87 (13.50)	1.03 (0.67, 1.58)	0.902
	TT/CT	324 (55.30)	362 (56.10)	1.08 (0.82, 1.43)	0.57
rs6691117	AA	288 (49.10)	342 (52.90)	1.00 (Reference)	
	AG	232 (39.60)	255 (39.40)	0.93 (0.69, 1.24)	0.619
	GG	66 (11.30)	50 (7.70)	1.27 (0.77, 2.08)	0.343
	GG/AG	298 (50.90)	305 (47.10)	0.99 (0.75, 1.30)	0.933
rs7525160	GG	180 (30.70)	258 (39.90)	1.00 (Reference)	
	CG	286 (48.80)	296 (45.70)	1.37 (1.01, 1.86)	0.047*
	CC	120 (20.50)	93 (14.40)	1.69 (1.13, 2.54)	0.011*
	CC/CG	406 (69.30)	389 (60.10)	1.46 (1.09,1.95)	0.012*

a Adjusted for gender, smoking, Hepatitis B and drinking status. *P < 0.05, statically significant.

### Stratified Analysis of Demographic Characteristics and Environmental Factors

We analyzed the polymorphism of *CD35* rs7525160 and HCC risk, based on factors including demographic characteristics (gender, age) and environmental factors (hepatitis B, family history of liver cancer, drinking, and smoking status); (**Supplementary Table S4**). Stratified analysis indicated that the *CD35* rs7525160 CC/CG genotype increased the risk of HCC in patients younger than 65 years (*P* for interaction=0.042; adjusted OR=1.85; 95% *CI* [1.30–2.62]; *P*=0.001).

### Genotype Frequency and Effects on Tumor Clinicopathological Types

Further stratified analysis of the clinical characteristics of HCC patients revealed that the *CD35* rs7525160 CC/CG genotype may increase the risk of different clinicopathological types of HCC, especially among patients with AFP ≥ 400 ng/ml [adjusted OR=1.94; 95% *CI* (1.24–3.04); *P*=0.004], tumor size > 5 cm [adjusted OR=1.75; 95% *CI* (1.19–2.56); *P*=0.004], TNM stage III/IV [adjusted OR=1.64; 95% *CI* (1.07–2.52); *P*=0.024], background cirrhosis [adjusted OR=1.50; 95% *CI* (1.05–2.15); *P*=0.026], or portal vein tumor thrombosis [adjusted OR=2.30; 95% *CI* (1.24–4.27); *P*=0.008] (**Supplementary Table S5**).

### CD35 Genetic Variation on Postoperative Recurrence of HCC and OS Analysis

**Figure 1:**
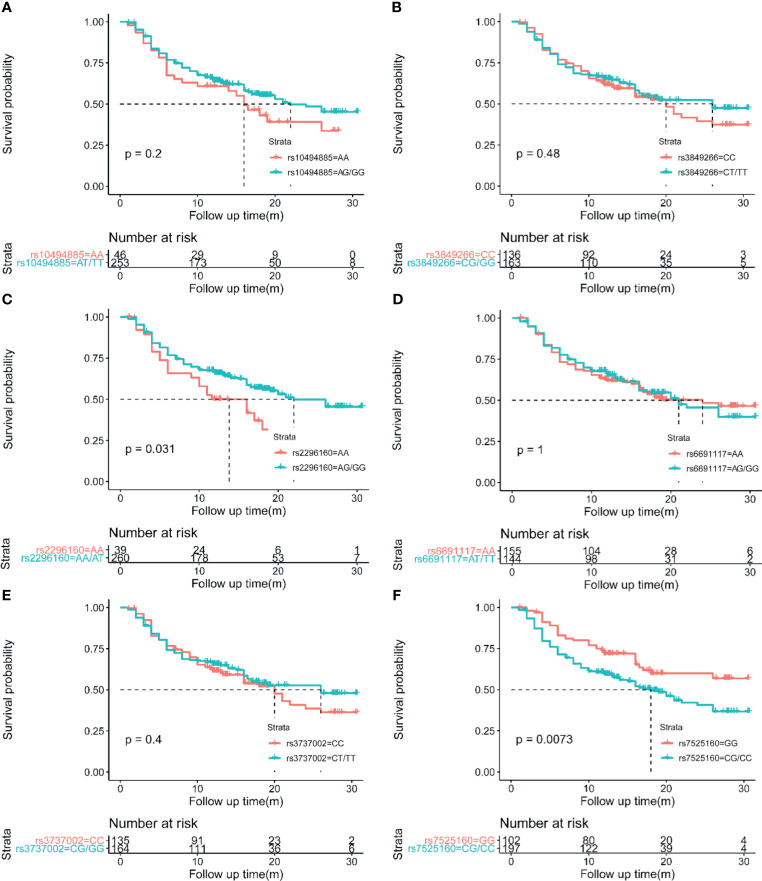
Kaplan-Meier analyses of CD35 genetic variation on postoperative HCC recurrence. **(A)** CD35 rs10494885. **(B)** CD35 rs3849266. **(C)** CD35 rs2296160. **(D)** CD35 rs6691117. **(E)** CD35 rs3737002. **(F)** CD35 rs7525160.

As mentioned above, *CD35* rs7525160 resulted in increased HCC risk, especially in advanced stage or big tumors, suggesting its potential predictive value in prognosis. To evaluate this, we detected the effects of six *CD35* SNP genotypes on the recurrence rate and mean recurrence-free survival (MRFS) of 299 HCC patients who underwent curative hepatectomy. Patients who were treated with transcatheter arterial chemoembolization (TACE), radiofrequency ablation, or drugs only were excluded to eliminate the influence of different treatments on the prognosis of HCC. Kaplan-Meier analyses showed that the MRFS was 17.82 months with 95% *CI* of 16.104–19.544 months for *CD35* rs7525160 CC/CG individuals, which was shorter than that for individuals carrying the GG genotype (MRFS 22.05 months; 95% *CI* [19.819–24.289 months]; *P*=0.0073); (**Figure 1**).

**Figure 2:**
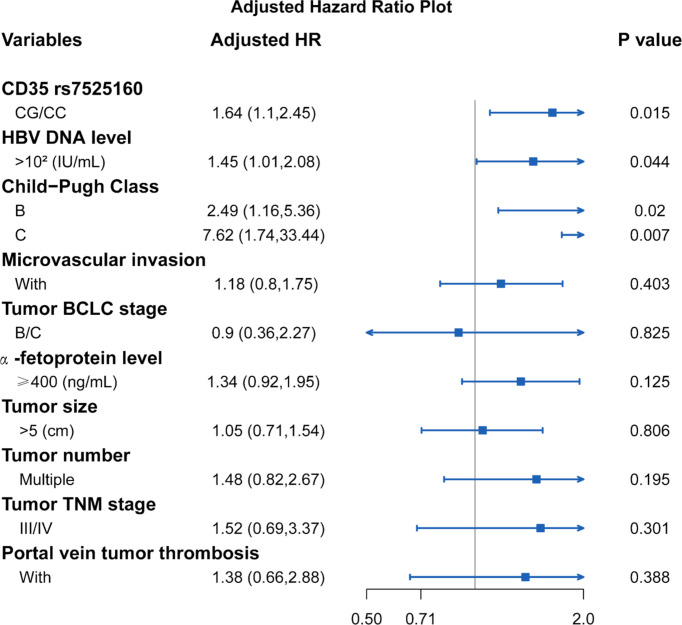
Cox analysis of potential factors for recurrence analysis in patients with resected HCC.

Considering that the recurrence of HCC is related to many clinical factors, we performed univariate survival analysis to identify possible factors affecting the recurrence of HCC in hepatectomy patients. Our result showed that HBV DNA level ≥10^2^ IU/mL [HR=1.77; 95% *CI* (1.26,2.47); *P*=0.011], Child-Pugh Class B [HR=2.55; 95% *CI* (1.24,5.22); *P*=0.011], Child-Pugh Class C [HR=6.56; 95% *CI* (1.60,26.84); *P*=0.009], microvascular invasion [HR=1.68; 95% *CI* (1.18,2.38); *P*=0.004], tumor BCLC stage B/C [HR=2.17; 95% *CI* (1.52,3.08); *P*<0.001], AFP level ≥ 400 ng/mL [HR=1.68; 95% *CI* (1.19,2.38); *P*=0.003], tumor size > 5 cm [HR=1.55; 95% *CI* (1.11,2.17); *P*=0.014], multiple tumor number [HR=1.62; 95% *CI* (1.10,2.39); *P*=0.014], TNM tumor stage III/IV [HR=2.47; 95% *CI* (1.73,3.52); *P*<0.001], and portal vein tumor thrombosis [HR=2.44; 95% *CI* (1.58,3.76); *P*<0.001] were factors that were possibly associated with HCC recurrence (**Table 3**). The Cox proportional hazard ratio model was used to analyze the effects of the *CD35* rs7525160 genotype on HCC recurrence in patients undergoing hepatectomy. The analysis indicated that *CD35* rs7525160 remained a significant independent risk factor for postoperative recurrence of HCC (adjusted HR=1.64; 95% *CI* [1.10–2.45]; *P*=0.015); (**Table 4** and **Figure 2**). With regard to OS analysis, 27 of 299 patients (9.0%) had died between the median follow-up of 18.0 months and maximum follow-up time of 30.7 months. Univariate analyses of overall survival are summarized (**Supplementary Table S6**). No statistically significant association was found between the six Tag SNPs in *CD35* and OS.

**Table 3:** Univariate survival analysis of clinical factors associated with HCC recurrence.

Variable	n	Recur [n (%)]	HR (95%*CI*)	*P*-value
Age				
<65 years	235	112 (81.20%)	1	0.174
≥65 years	64	26 (18.8%)	0.74 (0.49,1.14)	
Gender				
Female	48	24 (17.4%)	1	0.473
Male	251	114 (82.6%)	0.85 (0.55,1.32)	
Hepatitis B				
Without	61	26 (18.8%)	1	0.571
With	238	112 (81.2%)	1.13 (0.74,1.73)	
Smoking status				
Never	138	62 (44.9%)	1	0.65
Ever/current	161	76 (55.1%)	1.08 (0.77,1.51)	
Drinking status				
Never	166	77 (55.8%)	1	0.857
Ever/current	133	61 (44.2%)	1.03 (0.74,1.44)	
HBV DNA level (IU/mL)				
<10^2^	168	67 (48.6%)	1	0.011*
≥10^2^	130	71 (51.4%)	1.77 (1.26,2.47)	
Child-Pugh Class				
A	286	128 (92.8%)	1	
B	11	8 (5.8%)	2.55 (1.24,5.22)	0.011*
C	2	2 (1.4%)	6.56 (1.60,26.84)	0.009*
Microvascular invasion				
Without	202	87 (63.5%)	1	0.004*
With	92	50 (36.5%)	1.68 (1.18-2.38)	
Tumor stage (BCLC)				
0/A	228	91 (65.9%)	1	< 0.001*
B/C	71	47 (34.1%)	2.17 (1.52,3.08)	
α-fetoprotein level(ng/mL)				
<400	208	85 (61.6%)	1	0.003*
≥400	91	53 (38.4%)	1.68 (1.19,2.38)	
Tumor size (cm)				
≤5cm	150	60 (43.5%)	1	0.011*
>5cm	149	78 (56.5%)	1.55 (1,11-2.17)	
Tumor number				
Single	244	104 (75.4%)	1	0.014*
Multiple	55	34 (24.6%)	1.62 (1.10,2.39)	
Tumor stage (TNM)				
I/II	233	92 (66.7%)	1	< 0.001*
III/IV	66	46 (33.3%)	2.47 (1.73,3.52)	
Background cirrhosis				
Absent	88	33 (23.9%)	1	0.166
Present	211	105 (76.1%)	1.32 (0.89,1.95)	
Portal vein tumor thrombosis				
No	265	113 (81.9%)	1	<0.001*
Yes	34	25 (18.1%)	2.44 (1.58,3.76)	
Distant metastasis				
No	297	137 (99.3%)	1	0.88
Yes	2	1 (0.7%)	1.16 (0.16,8.33)	

*P < 0.05, statically significant.

**Table 4:** Cox analysis of potential factors for recurrence analysis in patients with resected HCC.

Variables	Crude HR (95% CI)	Adjusted HR (95% CI)^a^	*P*-value (Wald’s test)
rs7525160			
GG	1	1	
CG/CC	1.67 (1.14,2.44)	1.64 (1.10,2.45)	0.015*
HBV DNA level (IU/mL)			
<10^2^	1	1	
>10^2^	1.77 (1.26,2.47)	1.45 (1.01,2.08)	0.044*
Child-Pugh Class (C vs. B/A)			
A	1	1	
B	2.55 (1.24,5.22)	2.49 (1.16,5.36)	0.020*
C	6.56 (1.6,26.84)	7.62 (1.74,33.44)	0.007*
Microvascular invasion			
Without	1	1	
With	1.68 (1.18,2.38)	1.18 (0.80,1.75)	0.403
Tumor stage (BCLC)			
0/A	1	1	
B/C	2.17 (1.52,3.08)	0.9 (0.36,2.27)	0.825
α-fetoprotein level (ng/mL)			
<400	1	1	
≥400	1.68 (1.19,2.38)	1.34 (0.92,1.95)	0.125
Tumor size (cm)			
≤5	1	1	
>5	1.55 (1.11,2.17)	1.05 (0.71,1.54)	0.806
Tumor number			
Single	1	1	
Multiple	1.62 (1.1,2.39)	1.48 (0.82,2.67)	0.195
Tumor stage (TNM)			
I/II	1	1	
III/IV	2.47 (1.73,3.52)	1.52 (0.69,3.37)	0.301
Portal vein tumor thrombosis			
Without	1	1	
With	2.44 (1.58,3.76)	1.38 (0.66,2.88)	0.388

*P < 0.05, statically significant. ^a^adjusted for gender, smoking, Hepatitis B and drinking status.

## Discussion

Chronic inflammation is a hallmark of cancer (31), while the complement cascade, which is implicated in immune and inflammatory responses, plays a pivotal role in tumorigenesis (24). CD35, an important component of innate immunity, not only plays a key role in the clearance of circulating immune complexes (32), but also inhibits the activity of C3 and C5 convertases in classical and alternative pathways and promotes their degradation to regulate complement cascade activation and inhibit the inflammatory response. Therefore, it is reasonable to postulate that genetic variants of CD35 in the complement system may confer susceptibility to cancer.

Several studies have confirmed that the *CD35* gene polymorphisms are closely related to a variety of malignant tumors, such as nasopharyngeal carcinoma, gallbladder cancer, bladder cancer, non-Hodgkin’s lymphoma, small cell lung cancer, stomach cancer, and ovarian cancer (24–26, 33). However, few studies have investigated the relationship between *CD35* polymorphisms and HCC. In our study, we demonstrated, for the first time, that one SNP (rs7525160) of six tag SNPs of *CD35* was associated with the risk of HCC in the Chinese Han population. Notably, compared with the GG genotype, the *CD35* rs7525160 CC/CG genotype was associated with an increased risk for developing HCC.

Diseases caused by genetic mutations usually occur at a relatively younger age; this is attributed to the vulnerability of youth to genetic effects rather than environmental exposure, as evidenced by several studies on young patients (34–36), indicating that gene-environmental interaction may enhance susceptibility to malignant tumors. Consistent with these data, our results also demonstrated that the *CD35* rs7525160 CC/CG genotype was a risk factor for HCC in patients younger than 65 years.

Although the recurrence and fatality rates of HCC have decreased with the popularization of screening among high-risk groups and the improvement of treatment regimens (7), HCC remains by far the second most common cause of cancer-related deaths worldwide (37). Numerous predictors related to the prognosis of liver cancer, such as AFP levels, tumor number, and tumor size (38), have been reported, but no effective indicators have been found to date. Studies have shown that SNPs may be useful for predicting the prognosis of many cancers (39). Further, our subgroup analysis revealed that genotype frequency of the C allele of *CD35* rs7525160 in HCC patients was significantly different from that in controls, especially in patients with AFP ≥ 400 ng/ml, tumor diameter of > 5 cm, TNM stage III/IV, cirrhosis, and portal vein thrombosis, which suggested that CD rs7525160 CC/CG genotype may be related to the type of HCC with poor prognosis. To eliminate the effect of different treatments on the prognosis for HCC, we only studied the 299 untreated patients who had undergone hepatectomy to analyze the association between *CD35* and RFS/OS. We found that the *CD35* rs7525160 CC/CG genotypes were significantly associated with a shorter mean RFS for HCC patients, and rs7525160 was identified as an independent predictor of RFS in HCC patients by Cox analysis. However, no relationship between *CD35* polymorphism and OS was found in this study, which may be due to OS and postoperative recurrence of HCC being affected by many factors, including the choice of treatment following recurrence and the economic status of patients, among others.

This study was beset by several limitations that need to be addressed. First, our results are based only on the Chinese Han population. However, the allele frequency patterns of *CD35* polymorphisms vary greatly between different ethnic groups. Therefore, our results cannot be applied to other populations, and data pertaining to multi-ethnic and multi-region populations of non-Chinese Han populations are felt to be required. Secondly, our study focused only on *CD35* polymorphism, HCC susceptibility and prognosis at the genetic level, and therefore, further functional verification needs to be considered. Thirdly, a larger sample size is needed, especially when investigating the association between genetic polymorphism and HCC prognosis.

## Conclusion

In summary, we found that *CD35* rs7525160 may be associated with increased HCC susceptibility in the Chinese Han population, especially in individuals younger than 65 years. In addition, our results indicated that the *CD35* rs7525160 CC/CG genotype is closely related to a pathological type associated with poor prognoses for HCC and acts as an independent risk factor for a shorter RFS time following hepatectomy. Our results also suggest that *CD35* rs7525160 may be used as a biomarker for screening as well as for prognostic prediction of HCC. Additional well-designed studies with larger sample sizes and long-term follow-up periods, that encompass different ethnic groups, may be required to better elucidate the causal relationship between *CD35* polymorphism and HCC risk and prognosis.

## Data Availability Statement

The original contributions presented in the study are included in the article/**Supplementary Material**. Further inquiries can be directed to the corresponding author.

## Ethics Statement

The studies involving human participants were reviewed and approved by the Ethics Committee of Sichuan University (Ethics No. 2017-264). The patients/participants provided their written informed consent to participate in this study.

## Author Contributions

LmL and QL performed the research and wrote the manuscript. LmL and ZS were responsible for the conceptualization, investigation, and validation of the study. QL and ZS analyzed the data. LxL and BC contributed to the sample collection. YP and YB reviewed and edited the manuscript. FL was in charge of supervision, conceptualization, and project administration. All authors contributed to the article and approved the submitted version.

## Funding

This study was supported by the National Natural Science Foundation of China [grant numbers 81602910, 81702002], and the Sichuan Science and Technology Program [grant numbers 2019YFS0284, 2019YFS0287, 2019YFS0370].

## Conflict of Interest

The authors declare that the research was conducted in the absence of any commercial or financial relationships that could be construed as a potential conflict of interest.

## Publisher’s Note

All claims expressed in this article are solely those of the authors and do not necessarily represent those of their affiliated organizations, or those of the publisher, the editors and the reviewers. Any product that may be evaluated in this article, or claim that may be made by its manufacturer, is not guaranteed or endorsed by the publisher.

## Supplementary Material

Supplementary Figure 1 MALDI-TOF genotyping and direct sequencing map for CD35 rs7525160. **(A)** The three genotypes of CD35 rs7525160 polymorphism detected by MALDI-TOF. **(B)** Sequencing map for genotypes of CD35 rs7525160 GG genotype. **(C)** Sequencing map for genotypes of CD35 rs7525160 CG genotype.

## Abbreviations

HCC, hepatocellular carcinoma; MALDI-TOF-MS, matrix assisted laser desorption ionization time of flight mass spectrometry method; AFP, α-fetoprotein; BCLC, Barcelona-Clinic-Liver-Cancer; TNM, tumor–node–metastasis; AD, Alzheimer’s disease; MAF, minor allele frequency; CT, computed tomography; MRI, magnetic resonance imaging; RFS, Recurrence Free Survival; OS, Overall Survival; HWE, Hardy‐Weinberg equilibrium; OR, odds ratios; CI, confidence intervals; MRFS, mean recurrence-free survival; TACE, Transcatheter Arterial Chemoembolization; SNP, Single Nucleotide Polymorphism; Tag SNP, Tag Single Nucleotide Polymorphism; HBV, Hepatitis B Virus; HCV, Hepatitis C Virus.
