# Supplementary figures and images for: Genetic Polymorphisms in CD35 Gene Contribute to the Susceptibility and Prognosis of Hepatocellular Carcinoma
Source: Front Oncol. 2021 Aug 5;11:700711. doi: 10.3389/fonc.2021.700711 (PMC8374953; doi:10.3389/fonc.2021.700711)

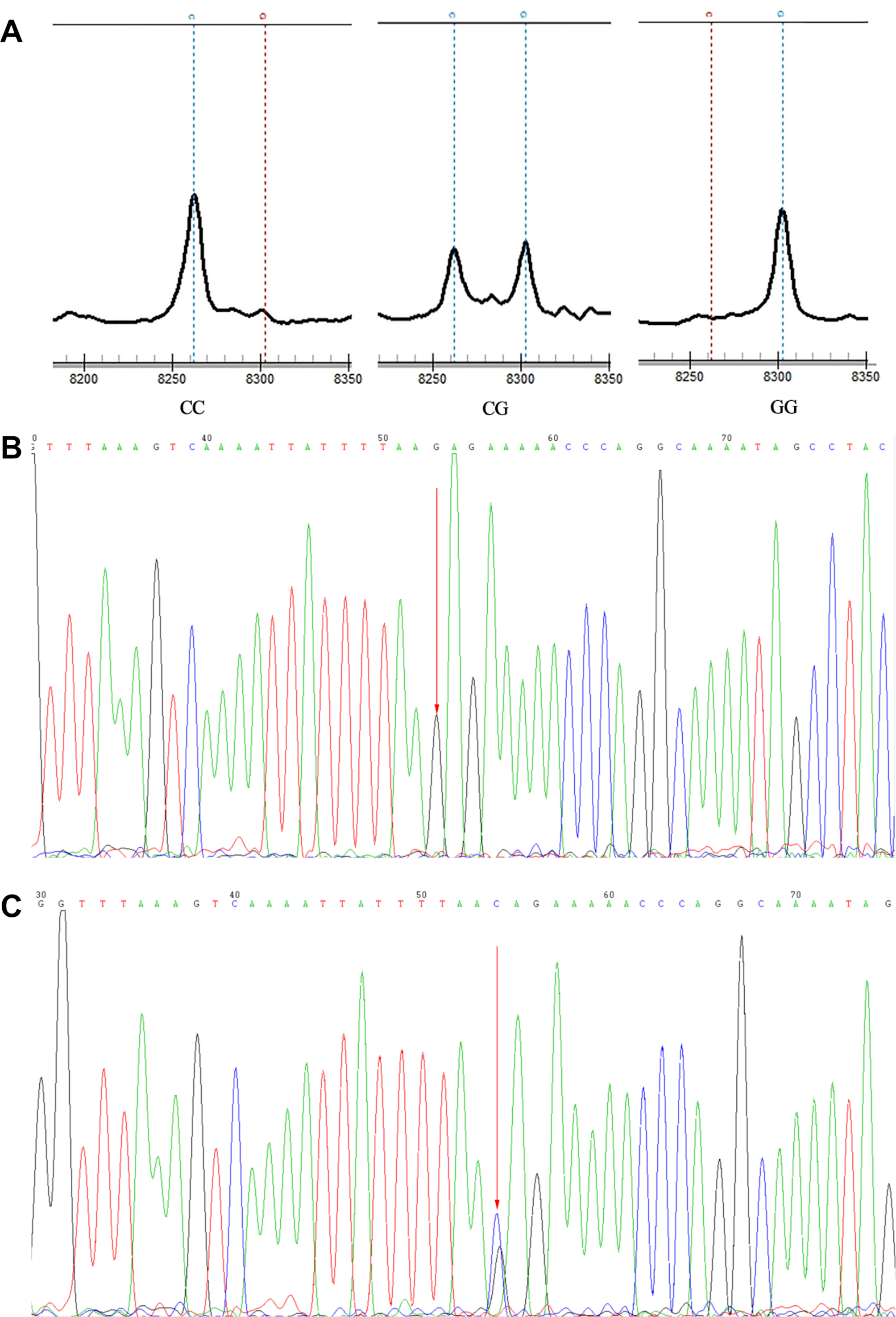

Supplement: Supplementary Figure 1 — MALDI-TOF genotyping and direct sequencing map for CD35 rs7525160. (A) The three genotypes of CD35 rs7525160 polymorphism detected by MALDI-TOF. (B) Sequencing map for genotypes of CD35 rs7525160 GG genotype. (C) Sequencing map for genotypes of CD35 rs7525160 CG genotype. [file Image_1.tif]
