# Supplementary material for: Genetic Polymorphisms in CD35 Gene Contribute to the Susceptibility and Prognosis of Hepatocellular Carcinoma
Source: Front Oncol. 2021 Aug 5;11:700711. doi: 10.3389/fonc.2021.700711 (PMC8374953; doi:10.3389/fonc.2021.700711)
Supplement: Supplementary file 2 [file Table_1.docx]

**Supplementary Table S1.** SNPs of CD35 gene evaluated in this study

| **SNP ID** | **Base change** | **Chromosome** | **MAF in controls** | ***P* for HWE** | **Functional Consequence** |
| --- | --- | --- | --- | --- | --- |
| rs10494885 | A to G | 1q32 | 0.14 | 0.10 | Downstream transcript variant |
| rs2296160 | A to G | 1q32 | 0.12 | 0.61 | Missense variant, coding sequence variant |
| rs3737002 | C to T | 1q32 | 0.13 | 0.15 | Missense variant, coding sequence variant |
| rs3849266  rs6691117  rs7525160 | C to T  A to G  G to C | 1q32  1q32  1q32 | 0.13  0.08  0.14 | 0.09  0.95  0.42 | Intron variant  Coding sequence variant, missense variant  Upstream transcript variant |

Abbreviations: SNP, single nucleotide polymorphism; MAF, minor allele frequency; HWE, Hardy-Weinberg equilibrium.
