# Supplementary material for: Genetic Polymorphisms in CD35 Gene Contribute to the Susceptibility and Prognosis of Hepatocellular Carcinoma
Source: Front Oncol. 2021 Aug 5;11:700711. doi: 10.3389/fonc.2021.700711 (PMC8374953; doi:10.3389/fonc.2021.700711)
Supplement: Supplementary file 3 [file Table_2.docx]

**Supplementary Table S2.** Primer sequences used in the selected tag SNPS of CD35 genotyping

| **SNP ID** | **PCR primer 1 (5’ to 3’)** | **PCR primer 2 (5’ to 3’)** | **Extension Primer (5’ to 3’)** |
| --- | --- | --- | --- |
| rs10494885 | ACGTTGGATGCCAGCCAACTGACCTTTATG | ACGTTGGATGTGATGTGTAATGCCACAGAC | cGTCTGATTTTCTTTCCTGTTAC |
| rs2296160 | ACGTTGGATGAGATGCCAGAATTCCTCAGC | ACGTTGGATGCCAGAGTGATGTTTTGTGAC | aAACTATGAGAGCATCATGTG |
| rs3737002 | ACGTTGGATGAAATACCCAGGACGGCATTC | ACGTTGGATGCAGAGCAGTTTCCATTTGCC | aaaaTCAAAGTCATTAATTGGGATC |
| rs3849266  rs6691117  rs7525160 | ACGTTGGATGATAACTCGCCCCTCAATCTG  ACGTTGGATGACCCTACCATGACAAACCCG  ACGTTGGATGCAAAATCAAGGTTTAAAGTC | ACGTTGGATGGCAGGCAACTGTGTTTATAC  ACGTTGGATGGAGTACCAGGAAACAGGAGT  ACGTTGGATGTCCTTCTGACATGTACTGCC | CCTCAATCTGCATTGATCCA  GGCTGACATCTAAATCTGA  AAGGTTTAAAGTCAAAATTATTTTAA |
