# Supplementary material for: Genetic Polymorphisms in CD35 Gene Contribute to the Susceptibility and Prognosis of Hepatocellular Carcinoma
Source: Front Oncol. 2021 Aug 5;11:700711. doi: 10.3389/fonc.2021.700711 (PMC8374953; doi:10.3389/fonc.2021.700711)
Supplement: Supplementary file 4 [file Table_3.docx]

**Supplementary Table S3.** Clinical and pathological features of HCC patients treated with hepatectomy

| **Covariate** | **n** | **rs7525160 genotype** | | ***P*-value** |
| --- | --- | --- | --- | --- |
|  |  | **CG/CC** | **GG** |  |
| Age |  |  |  | 0.428 |
| < 65 years | 235 | 158 | 77 |  |
| ≥ 65 years | 64 | 39 | 25 |  |
| Gender |  |  |  | 1.198 |
| Female | 48 | 36 | 12 |  |
| Male | 251 | 161 | 90 |  |
| Hepatitis B |  |  |  | 0.925 |
| Without | 61 | 41 | 20 |  |
| With | 238 | 156 | 82 |  |
| Smoking status |  |  |  | 0.116 |
| Never | 138 | 84 | 54 |  |
| Ever/current | 161 | 113 | 48 |  |
| Drinking status |  |  |  | 0.782 |
| Never | 166 | 111 | 55 |  |
| Ever/current | 133 | 86 | 47 |  |
| HBV DNA |  |  |  |  |
| < 10^2^ | 168 | 107 | 61 | 0.461 |
| ≥ 10^2^ | 130 | 89 | 41 |  |
| Child-Pugh Class |  |  |  |  |
| A | 2 | 0 | 2 | 0.101 |
| B | 286 | 191 | 95 |  |
| C | 11 | 6 | 5 |  |
| Microvascular invasion |  |  |  |  |
| Without | 202 | 127 | 75 | 0.095 |
| With | 93 | 68 | 25 |  |
| Tumor stage (BCLC) |  |  |  |  |
| 0/A | 228 | 148 | 80 | 0.622 |
| B/C | 71 | 49 | 22 |  |
| α-fetoprotein level (ng/mL) |  |  |  |  |
| < 400 | 91 | 69 | 22 | 0.024 |
| ≥ 400 | 208 | 128 | 80 |  |
| Tumor size (cm) |  |  |  |  |
| ≤ 5cm | 149 | 103 | 46 | 0.291 |
| >5cm | 150 | 94 | 56 |  |
| Tumor number |  |  |  |  |
| Single | 244 | 162 | 82 | 0.816 |
| Multiple | 55 | 35 | 20 |  |
| Tumor stage (TNM) |  |  |  |  |
| I/II | 233 | 150 | 83 | 0.375 |
| III/IV | 66 | 47 | 19 |  |
| Background cirrhosis |  |  |  |  |
| Present | 88 | 60 | 28 | 0.684 |
| Absent | 211 | 137 | 74 |  |
| Portal vein tumor thrombosis |  |  |  |  |
| No | 265 | 170 | 95 | 0.115 |
| Yes | 34 | 27 | 7 |  |
| Distant metastasis |  |  |  |  |
| No | 297 | 196 | 101 | 1.000 |
| Yes | 2 | 1 | 1 |  |
