# Supplementary material for: Genetic Polymorphisms in CD35 Gene Contribute to the Susceptibility and Prognosis of Hepatocellular Carcinoma
Source: Front Oncol. 2021 Aug 5;11:700711. doi: 10.3389/fonc.2021.700711 (PMC8374953; doi:10.3389/fonc.2021.700711)
Supplement: Supplementary file 5 [file Table_4.docx]

**Supplementary Table S4.** Stratified analysis of CD35 rs7525160 genetic variation and HCC risk

| **Variable** | **n** | **HCC (n)** | **Adjusted OR (95% CI) ^a^** | ***P*-value** | ***P* for interaction** |
| --- | --- | --- | --- | --- | --- |
| Age  < 65 years  ≥ 65 years  Gender  Female  Male | 960  273  445  788 | 444  142  95  491 | 1.85 (1.30-2.62)  0.98 (0.56-1.74)  1.68 (0.94-2.97)  1.37 (0.96-1.94) | 0.001*  0.954  0.078  0.080 | 0.042*  0.336 |
| Hepatitis B  Without  With  Smoking status  Never  Ever/current | 648  585  781  452 | 144  442  275  311 | 1.38 (0.89-2.14)  1.69 (1.13-2.52)  1.42 (0.98-2.08)  1.57 (0.97-2.55) | 0.151  0.010*  0.065  0.069 | 0.324  0.695 |
| Drinking status  Never  Ever/current  Family history of liver cancer  Without  With | 817  416  1185  48 | 326  260  560  26 | 1.50 (1.03-2.18)  1.50 (0.92-2.45)  1.49 (1.10-2.02)  3.04 (0.67-13.75) | 0.034*  0.104  0.010*  0.148 | 0.865  0.640 |

^a^ adjusted for gender, smoking, Hepatitis B and drinking status. **P* < 0.05, statically significant.
