# Supplementary material for: Genetic Polymorphisms in CD35 Gene Contribute to the Susceptibility and Prognosis of Hepatocellular Carcinoma
Source: Front Oncol. 2021 Aug 5;11:700711. doi: 10.3389/fonc.2021.700711 (PMC8374953; doi:10.3389/fonc.2021.700711)
Supplement: Supplementary file 6 [file Table_5.docx]

**Supplementary Table S5.** Subgroup analyses of CD35 rs7525160 genetic variation and HCC risk

| **Characteristics** | **GG [n (%)]** | **CC/CG [n (%)]** | **Adjusted OR (95% CI) ^a^** | ***P*-value** |
| --- | --- | --- | --- | --- |
| Tumor size (cm)  ≤ 5cm  > 5cm | 112 (62.20)  68 (37.80) | 223 (54.90)  183 (45.10) | 1.37 (0.98-1.92)  1.75 (1.19-2.56) | 0.067  0.004* |
| Tumor number  Single  Multiple  Tumor stage (TNM)  I/II  III/IV  Liver cirrhosis | 117 (65.00)  63 (35.00)  131 (72.80)  49 (27.20) | 273 (67.20)  133 (32.80)  283 (69.70)  123 (30.30) | 1.55 (1.11-2.15)  1.45 (0.97-2.17)  1.47 (1.07-2.03)  1.64 (1.07-2.52) | 0.009*  0.073  0.018*  0.024* |
| With  Without  Portal vein rumor thrombus  With  Without  Distant metastasis  With  Without  α-fetoprotein level (ng/mL)  < 400  ≥ 400 | 104 (57.80)  76 (42.20)  17 (9.40)  163 (90.60)  5 (2.80)  175 (97.20)  137 (76.10)  43 (23.90) | 244 (60.10)  162 (39.90)  61 (15.00)  345 (85.00)  14 (3.40)  392 (96.60)  281 (69.20)  125 (30.80) | 1.50 (1.05-2.15)  1.45 (1.01-2.08)  2.30 (1.24, 4.27)  1.44 (1.06, 1.95)  1.86 (0.63,5.50)  1.50 (1.12,2.02)  1.40 (1.02, 1.92)  1.94 (1.24, 3.04) | 0.026*  0.042*  0.008*  0.018*  0.264  0.007*  0.035*  0.004* |

^a^ adjusted for gender, smoking, Hepatitis B and drinking status. **P* < 0.05, statically significant.
