# Supplementary material for: Genetic Polymorphisms in CD35 Gene Contribute to the Susceptibility and Prognosis of Hepatocellular Carcinoma
Source: Front Oncol. 2021 Aug 5;11:700711. doi: 10.3389/fonc.2021.700711 (PMC8374953; doi:10.3389/fonc.2021.700711)
Supplement: Supplementary file 7 [file Table_6.docx]

**Supplementary Table S6.** CD35 genetic variation on overall survival of hepatectomy HCC patients

| **CD35 SNP ID** | **Genotype** | **Frequency [n (%)]** | **Death [n (%)]** | **MST (months)** | **95% CI (months)** | ***P*-value** |
| --- | --- | --- | --- | --- | --- | --- |
| rs10494885  rs2296160  rs3737002  rs3849266  rs6691117  rs7525160 | AA  GG/AG  AA  GG/AG  CC  TT/CT  CC  TT/CT  AA  GG/AG  GG  CC/CG | 46 (15.4)  253 (84.6)  39 (13.0)  260 (87.0)  135 (45.2)  164 (54.8)  136 (45.5)  163 (54.5)  155 (51.8)  144 (48.2)  102 (34.1)  197 (65.9) | 4 (14.8)  23 (85.2)  2 (7.4)  25 (92.6)  11 (40.7)  16 (59.3)  11 (40.7)  16 (59.3)  13 (48.1)  14 (55.2)  7 (25.9)  20 (74.1) | 28.706  28.535  29.310  28.457  28.667  28.448  28.691  28.432  28.684  28.467  28.891  28.350 | 26.835-30.576  27.688-29.382  27.428-31.192  27.618-29.295  27.516-29.817  27.395-29.501  27.553-29.829  27.371-29.492  27.631-29.737  27.350-29.584  27.669-30.112  27.369-29.331 | 0.876  0.383  0.730  0.693  0.718  0.356 |
